# Hereditary Thrombophilia in the Era of COVID-19

**DOI:** 10.3390/healthcare10060993

**Published:** 2022-05-27

**Authors:** Oana Viola Badulescu, Paul Dan Sirbu, Nina Filip, Gabriela Bordeianu, Elena Cojocaru, Cristian Constantin Budacu, Minerva Codruta Badescu, Iris Bararu-Bojan, Bogdan Veliceasa, Manuela Ciocoiu

**Affiliations:** 1 Department of Pathophysiology, Morpho-Functional Sciences (II), Faculty of Medicine, “Grigore T. Popa” University of Medicine and Pharmacy, 700115 Iasi, Romania; violabadulescu@yahoo.com (O.V.B.); iris_bararu@yahoo.com (I.B.-B.); mciocoiu2003@yahoo.com (M.C.); 2 Department of Orthopedics and Traumatology, Surgical Science (II), Faculty of Medicine, “Grigore T. Popa” University of Medicine and Pharmacy, 700115 Iasi, Romania; pdsirbu@yahoo.com (P.D.S.); velbogdan@yahoo.com (B.V.); 3 Department of Biochemistry, Morpho-Functional Sciences (II), Faculty of Medicine, “Grigore T. Popa” University of Medicine and Pharmacy, 700115 Iasi, Romania; gabrielabordeianu@yahoo.co.uk; 4 Department Morpho-Functional Sciences (I), Faculty of Medicine, “Grigore T. Popa” University of Medicine and Pharmacy, 700115 Iasi, Romania; ellacojocaru@yahoo.com; 5 Department of Dentoalveolar and Maxillofacial Surgery, Faculty of Dental Medicine, “Grigore T. Popa” University of Medicine and Pharmacy, 700115 Iasi, Romania; cristibudacu@yahoo.com; 6 Department of Internal Medicine, Faculty of Medicine, “Grigore T. Popa” University of Medicine and Pharmacy, 700115 Iasi, Romania

**Keywords:** hereditary thrombophilia, coronavirus disease 2019, thrombosis, genetic profile

## Abstract

Thrombophilia, also called hypercoagulability or prothrombotic condition, usually reflects a certain imbalance that occurs either in the coagulation cascade or in the anticoagulation/fibrinolytic system. A similar imbalance may be induced by severe acute respiratory syndrome coronavirus 2 (SARS-CoV-2). Thrombotic complications are associated with multiorgan failure and increased mortality. In this context, activation of coagulation and thrombocytopenia appeared as prognostic markers in COVID-19. Our work provides a structured and updated analysis of inherited thrombophilia and its involvement in COVID-19, emphasizing the importance of diagnosing and initiating thromboprophylaxis. Since the state of hypercoagulation is directly correlated with COVID-19, we consider that studies on the genetic profiles of proteins involved in thrombophilia in patients who have had COVID-19 and thrombotic events are of great importance, both in treating and in preventing deaths due to COVID-19.

## 1. Introduction

Thrombophilia is a coagulation abnormality that increases the risk of thrombosis (the formation of thrombi in the blood vessels); it is also called hypercoagulability or prothrombotic condition. It usually reflects a certain imbalance occurring either in the coagulation cascade or in the anticoagulation/fibrinolytic system [1]. Severe acute respiratory syndrome coronavirus 2 (SARS-CoV-2), among other viral infections, may induce a similar imbalance. Thrombophilia can be inherited or acquired. The former is due to deficiencies of natural anticoagulants (antithrombin, protein C and protein S), increased homocysteine values, and changes in fibrinogen and coagulation factors. One important thing to note is that hereditary thrombophilia increases the risk of miscarriage. Acquired thrombophilia occurs as a result of secondary diseases, such as autoimmune disorders (antiphospholipid syndrome), trauma, or malignancy [1,2,3,4].

Thrombophilia aside, there are numerous (extrinsic) factors that increase the risk of thromboembolic disorders: obesity, surgery, smoking, pregnancy, the use of contraceptives, hormone replacement therapy, malignancy and antiphospholipid antibodies (lupus anticoagulant, anticardiolipin antibody, and anti-β2-glycoprotein-1 antibody) [5,6,7,8,9,10].

In-depth knowledge of all risk factors and understanding of their roles in the pathophysiology of venous thrombosis is of great importance, especially in the current pandemic context.

COVID-19 was initially described as an acute respiratory syndrome; later, other important features became apparent, in particular cytokine storms and systemic thromboembolism [11,12]. Since the beginning of the pandemic, significant efforts have been made worldwide to control and treat this disease. In the fight against the virus, the body over activates the immune system and causes a storm of cytokines [11]. The main components of a cytokine storm are the critical immune elements of the pro-inflammatory milieu.

Numerous studies have reported thrombosis as one of the most serious complications that can occur in patients with COVID-19 [13,14,15]. Thrombotic complications, such as deep-vein thrombosis, pulmonary embolism and stroke, are associated with multiorgan failure and increased mortality. In this context, activation of coagulation and thrombocytopenia have appeared as prognostic markers in COVID-19. Severe thrombotic manifestations of COVID-19 are thought to be due to the ability of severe acute respiratory syndrome coronavirus 2 to invade endothelial cells through the angiotensin-converting enzyme 2 (ACE-2). The cells, tissues and organs most affected are those with high expression of ACE-2, the entry receptor for severe acute respiratory syndrome coronavirus 2. Comorbidities that increase the risk of severe COVID-19 infection, including old age, obesity, diabetes, high blood pressure, respiratory disease, a compromised immune system, and coronary heart disease, or heart failure are associated with endothelial dysfunctional [16].

As potential therapies for the prevention of thrombosis associated with COVID-19, antithrombotic drugs, including heparin, factor XII inhibitors, and fibrinolytic drugs, have been administered [17]. One guide to thromboprophylaxis in COVID-19 recommends routine doses of thromboprophylaxis in the absence of contraindications in the hospital, increased intensity thromboprophylaxis in the intensive care unit, and consideration of anticoagulant thromboprophylaxis in patients with increased risk of venous thromboembolism post-hospital [18].

Improving diagnostic and treatment strategies can reduce thrombotic complications and ultimately improve the prognosis of patients with COVID-19.

We aimed to review available data on severe acute respiratory syndrome coronavirus 2 infections and inherited thrombophilia and to identify and highlight points relevant to therapeutic options in order to reduce mortality.

## 2. Blood Clotting Process

**Figure 1:**
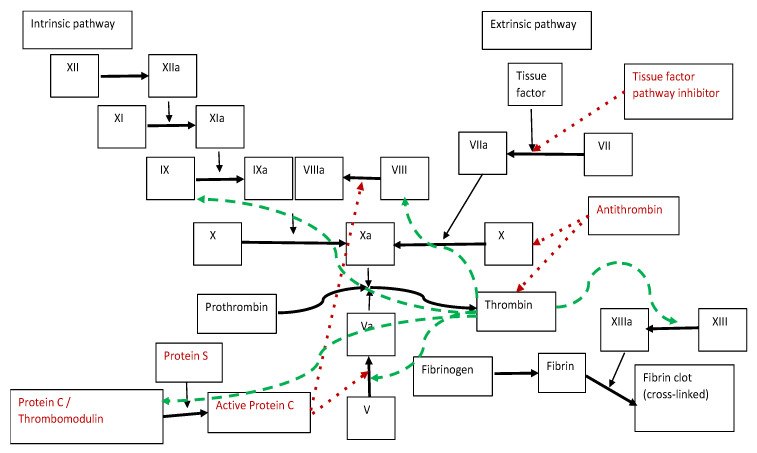
Schematic representation of the coagulation process: coagulation factors (in black) and natural anticoagulants (in red); red dotted line—inhibitory effect; green dashed line—stimulatory effect; thick line—chemical transformation; thin line—catalytic effect.

In essence, the phenomenon of blood clotting is the transformation of fibrinogen into insoluble fibrin [19,20] (Figure 1).

Classically, it is considered that the activation of the coagulation cascade can be initiated via two pathways: extrinsic and intrinsic. The extrinsic pathway is initiated by a tissue trauma that leads to damage to the vascular wall. The intrinsic pathway is initiated by exposure of the blood to a negatively charged surface or to collagen in the traumatized vascular wall [21,22]. Both pathways lead to the activation of factor X (following the cleavage of a link between arginine and isoleucine) and converge in a common end pathway [23].

## 3. Overview of COVID-19

Coronaviruses are single-stranded RNA viruses that have been known for over 50 years. They cause severe acute respiratory syndrome, which in many cases leads to death. The virus that causes COVID-19 is thought to have originated in bats and then spread to humans [24].

Coronavirus disease 2019, also known as COVID-19, is a real challenge for specialists. COVID-19 infection has affected the lives of people around the world in terms of both physical and mental health as well as financial implications. It is characterized by a high mortality rate worldwide [25]. Over this period, considerable efforts have been made to quickly diagnose the disease and prevent and treat it. COVID-19 is known to bind to the angiotensin-converting enzyme 2 receptor and enter cells through the mechanism of endocytosis. Real-time PCR (RT-PCR) is considered the gold standard for diagnosis. Measurement of plasma D-dimer levels has been put forward as a prognostic marker.

Since December 2019, it has been reported that COVID-19 has caused the majority of cases of pneumonia [26]. The average incubation period of the virus was reported to be 5.2 days. The most common initial symptoms are fever, cough and fatigue [27,28]. It is known that the main target of COVID-19 infection is the lung. Although the main target of infection is the lung, the wide distribution of ACE-2 receptors in other organs may lead to cardiovascular, gastrointestinal, kidney, liver, central nervous system and ocular damage. Numerous complications associated with COVID-19 presented in the literature are summarized in Table 1.

The course of COVID-19 disease in children is generally asymptomatic or mild compared to that seen in adults, for reasons that have not yet been clearly elucidated [61]. Most children have a low prevalence of symptoms [62]. The most common symptoms in children are fever, conjunctivitis, rash, and vomiting [63,64,65,66,67].

Although mortality is low in children with COVID-19, comorbidities are associated with a more severe course of infection [66,68,69,70].

It is obvious that COVID-19 infection causes severe coagulation disorders and that these play a crucial role in the outcomes of patients. Regarding COVID-19, the pathological changes of the disease and its pathogenesis in humans have not been clearly explained to date. Dissemination of information is key, including timely publication of statistics data, early diagnosis, reporting, and isolation and treatment. What is certain is that the lungs are the major organ involved.

## 4. Inherited Thrombophilia

Hereditary thrombophilia occurs when an inherited factor requires interaction with components that are inherited or acquired prior to a clinical disorder [71].

The most common causes of hereditary thrombophilia are: antithrombin deficiency, protein C deficiency, protein S deficiency, disturbances in fibrinogen levels, elevated homocysteine levels, factor II mutation (F2 c.*97G > A; previous nomenclature G20210A) and factor V Leiden mutation (HGVS nomenclature: F5 c.1601G > A) [71].

### 4.1. Antithrombin Deficiency

Antithrombin (AT) is a thrombin inhibitor and belongs to the serpin family (serine protease inhibitors); these are plasma proteins that inhibit serine proteases to prevent their uncontrolled activity. AT binds to the catalytic site of thrombin and is cleaved, and the resulting fragments remain firmly attached, block the active site and no longer allow fibrinogen to bind. AT activity is potentiated by heparin [72]. The electrostatic interaction between the electronegative heparin groups and the electropositive AT groups (protonated lysine residues) causes a conformational change of the AT, resulting in an increase in its antithrombin activity by about 1000 times. AT also inactivates other serine proteases involved in coagulation, such as factors Xa, XIa, XIIa and kallikrein [72].

AT deficiency is classified into:

- Type I deficiency: reduced synthesis of the molecule (both antigenic and functional activity of AT in the blood are reduced);
- Type II deficiency: molecular defect (AT immunological activity is normal but functional activity is reduced);
- Type III: affected interaction between AT and heparin [71].

In elderly patients with antithrombin deficiency, the risk of thrombosis is increased and long-term administration of anticoagulants becomes unavoidable. Additionally, the incidence of venous thromboembolism is increased in pregnant women with AT deficiency [71]. Patients with type II AT deficiency have a higher risk of developing venous thromboembolism compared to those with type I AT deficiency [71].

### 4.2. Protein C Deficiency

Protein C is a protease produced by the liver that contains γ-carboxy-glutamate residues, formed with the participation of vitamin K. It is activated by thrombin. Attached to endothelial thrombomodulin, thrombin modifies its substrate specificity and activates protein C by proteolysis. Activated protein C, in the presence of a cofactor (protein S), degrades factors Va and VIIIa into inactive peptides, leading to coagulation limitation [72].

Hereditary deficiency is caused by a mutation in the PROC gene located on chromosome 2q14.3. Heterozygous and acquired deficiencies are more common than homozygous deficiencies [73]. An inherited or acquired risk factor for thrombophilia is protein C deficiency. People with inherited protein C deficiency have about a 2- to 11- fold increased risk of venous thromboembolism developing [72].

### 4.3. Protein S Deficiency

Protein S is a glycoprotein synthesized in the liver and is an important cofactor for protein C activation. Protein S is involved in the coagulation process, having anticoagulant properties both dependent on and independent of active protein C [73]. Approximately 40% of S protein circulates freely in the plasma and 60% with the plasma C4b binding protein (C4b-BP) [73].

Protein S deficiency is a rare genetic disorder of blood coagulation that is caused by a variation in the PROS1 gene. This variation is inherited in an autosomal dominant manner. A recent study published by Juhl et al. reported an association between two novel variants of the S protein gene and protein S deficiency [74].

The following types of S protein deficiency are known:

- Type I: characterized by decreased activated protein C cofactor activity, low values of total S protein and free S protein;
- Type II: characterized by decreased activated protein C cofactor activity, normal values of total S protein and free S protein;
- Type III: characterized by decreased activated protein C cofactor activity, normal values of total S protein and low values of free S protein [73].

Patients with hereditary protein S deficiency have a high risk of venous thromboembolism recurrence. A retrospective study has shown that annual incidences of a first recurrence after the first episode of venous thromboembolism were 8.4% in protein S deficient patients [75].

### 4.4. Disturbances in Fibrinogen Levels

Fibrinogen is a glycoprotein with an elongated molecular shape, one of the least soluble in the protein system of the blood and is essential for the formation of fibrin clots. An increased risk of bleeding is associated with acquired and congenital fibrinogen disorders that can lead to a decreased concentration or altered fibrinogen function [76].

Thrombosis as a consequence of fibrinogen malfunction has been explained by two possible mechanisms: one involves abnormal thrombin binding to abnormal fibrin, leading to increased thrombin levels, and the second refers to the function of abnormal fibrin stimulation in fibrinolysis mediated by plasminogen activators [77]. Some patients with disturbed fibrinogen levels may experience thrombophilia and sometimes both bleeding and thromboembolism [76].

### 4.5. Elevated Homocysteine Levels

Homocysteine (Hcy) is a non-protein amino acid involved in the metabolism of methionine [78], produced by S-adenosylmethionine (SAM) and S-adenosylhomocysteine (SAH). Homocysteine metabolism involves several enzymes, namely: methionine synthase (MS), methylenetetrahydrofolate reductase (MTHFR), cystathionine β-synthase (CBS), methionine synthase reductase (MSR) and betaine-homocysteine S-methyltransferase (BHMT). Homocysteine can be converted to methionine by remethylation by MTHFR and MS requiring methylcobalamin (vitamin B12). The MTHFR mutation results in reduced enzymatic activity and, consequently, accumulation of homocysteine. Hcy can form cystathionine by condensation with serine, via trans-sulfuration, a CBS-catalyzed reaction dependent on vitamin B6 [79,80,81,82].

At the plasma level, four forms of homocysteine can be found, namely: free Hcy, protein-bound Hcy (S-linked and N-bound), oxidized forms of Hcy, and Hcy-thiolactone. Hyperhomocysteinemia (HHcy) is defined as a plasma level greater than 15 μmol/L [83]. HHcy may be due to genetic defects in the enzymes involved in the metabolism of homocysteine and associated vitamin deficiencies. Elevated homocysteine levels were observed in B12 deficiencies even when folate levels were normal. Only concomitant supplementation of B vitamins (Vitamin B6, Vitamin B12) and folic acid has been reported to be effective in reducing Hcy levels. To date, a large number of studies indicate that Hcy is an independent risk factor for cardiovascular disease and that there is a higher correlation between homocysteine and atherosclerosis levels than between cholesterol and atherosclerosis levels [78,83]. Hcy is thought to be associated with vascular dysfunction through the following mechanisms: generation of reactive species (ROS), triggering/maintaining the inflammatory response; initiation of thrombotic phenomena [80,84]. Endothelial dysfunction is one of the major events in the pathogenic mechanism of cardiovascular disease. In the evolution of this disease, inflammation is the trigger for the process [85,86,87]. Reactive species are among the compounds present at the site of the inflammation and play multiple roles, including as markers of the intensity of the lesion phenomenon, and molecules involved in defense and/or cell signaling. The literature indicates a link between homocysteine and reactive species. Hcy is a risk factor for thrombophilia and is associated with both venous thrombosis and arterial thrombosis [84]. A study by Ridker et al., over a period of 10 years, showed that the combination of hyperhomocysteinemia and Leiden factor V further increases the risk of venous thromboembolism [88].

### 4.6. Factor II Mutation

Prothrombin or coagulation factor II is the precursor of thrombin and is a major coagulation factor. It is synthesized in the liver and involves the participation of vitamin K. Factor II deficiency can be congenital or acquired.

A specific change in the genetic code causes the body to produce too much of the prothrombin protein. The most common point mutation of thrombosis is a factor II mutation known as prothrombin G20210A [89]. The F2 C.*97G > A (previous nomenclature G20210A) variant with autosomal dominant transmission is the second most common hereditary thrombophilia. Assuming that mutations in factor V Leiden and factor II are two genetic risk factors frequently involved in venous thromboembolism, Emmerich et al. [90] studied the risk of its occurrence in patients with both mutations. Their results showed that the frequency of Leiden factor V was lower in patients with pulmonary embolism than in patients with deep-vein thrombosis without pulmonary embolism, and the G20210A mutation was similar in both groups of patients [90].

### 4.7. Factor V Leiden

Factor V is a protein in the blood that is involved in the clotting cascade. The proteins involved in the coagulation cascade are activated when it is necessary to stop a hemorrhage. Anticoagulant proteins can deactivate factor V, stopping the formation of thrombi when coagulation is not required.

The point mutation in the factor V gene that leads to the replacement of arginine at position 506 with glutamine is responsible for the resistance to activated protein C. Factor V Leiden is an abnormal protein determined by a single-nucleotide polymorphism (1691G > A) in factor V, and is not susceptible to cleavage at position 506 by activated protein C [91]. Most people with factor V Leiden do not develop thrombophilia, but some may develop thrombosis that leads to long-term health problems and can be life-threatening. Persons with homozygous F2 c.*97G > A or double heterozygous carriers of factor V Leiden and F2 c.*97G > A do not have a risk of developing recurrent venous thromboembolism. Women may have an increased tendency to develop thrombophilia during pregnancy.

The most common inherited form of thrombophilia is related to factor V Leiden, and the second most common genetic form of thrombophilia is related to prothrombin and occurs in approximately 1.7–3% of the general European and American population [92].

## 5. A Closer Look at the Correlation with COVID-19

Many physicians treating COVID-19 patients have wondered whether patients with a hereditary thrombophilia develop COVID-19-associated coagulation disorders more frequently, whether these patients have a worse prognosis, and whether there are specific therapeutic options.

An important factor in patients diagnosed with COVID-19 is impaired coagulation function. A study of 49 patients with COVID-19 showed that AT levels were lower in non-survivors than in survivors. The authors reported that AT is strongly associated with mortality in COVID-19 and that this may be the link between obesity and a poorer prognosis in patients with COVID-19 [93]. Gardner et al. [94] tested AT levels in 19 patients with COVID-19 and reported an antithrombin deficiency in approximately 26% of them. In order to prevent death in patients with COVID-19, antithrombin deficiency should be considered.

Symptomatic subjects with hereditary protein S deficiency typically present with deep-vein thrombosis or pulmonary embolism [95]. In patients with COVID-19, free S protein may be reduced due to complications, leading to an increased risk of thromboembolism.

A study of the coagulation function of patients with SARS-CoV-2 reported elevated levels of D-dimer and fibrinogen. The results of the study showed significant hypercoagulability in patients with severe COVID-19 [96].

Similar results regarding hemostasis disorders in patients with COVID-19 were also reported by Hardy et al. [97], who investigated the following parameters: prothrombin time, heparin anti-Xa activity, fibrinogen, factor VIII activity, D-dimers, antithrombin and plasminogen activator inhibitor-I (PAI-1). In addition to hypercoagulability, the authors reported a low fibrinolytic capacity [97].

To date, there are very few studies on the link between inherited thrombophilia and COVID-19.

Studies showing the relationship between hereditary thrombophilia and COVID-19 are summarized in Table 2.

One study has been published investigating the possible correlation of thrombotic events with hereditary thrombophilia factors in patients who died of COVID-19 [104]. The authors evaluated the mutations in FV 506R/Q, MTHFR 223A/V, F2 20210G/A and PAI-1 4G/5G. The results obtained by them show that the highest percentage was detected in pulmonary artery thrombosis, followed by pulmonary embolism. Additionally, the incidence of MTHFR 223A/V heterozygous and PAI-1 4G/5G heterozygous genotypes was higher in patients with COVID-19 and thrombotic events, and that of FV 506R/Q and F2 20210G/A heterozygotes was lower. The authors state that studies are being performed on a larger number of patients in order to establish the relationship between hereditary thrombophilia factors and thrombosis associated with COVID-19 [104]. The MTHFR gene encodes 5,10-methylenetetrahydrofolate reductase, which is involved in homocysteine metabolism. The severity of COVID-19 could be associated with HHcy and possibly with depleted folic acid in infected cells [105,106,107]. HHcy was correlated with an increase in the incidence and severity of COVID-19 [108]. The results of the study conducted by Cappadona et al. [107] showed that evidence for an association with severe COVID-19 was found for five loci: two folate metabolic genes (MTHFR and MTR), two hemostasis inhibitor genes (PROC and ADAMTS13), and a thrombospondin gene (THBS2). HHcy is also a cause of hereditary thrombophilia.

A study conducted by de la Morena-Barrio et al. [99] on the involvement of hereditary thrombophilia in COVID-19 revealed that most patients with severe thrombophilia did not have thrombotic events during SARS-CoV-2 infection. The authors considered that the reason why the hypercoagulability condition did not lead to the development of new thrombotic episodes was related to the fact that these patients were already being treated with anticoagulant drugs before COVID-19 or at the beginning of the disease. In this context, long-term anticoagulation treatment at admission appears to protect patients with COVID-19 from the development of thrombosis [99].

On the other hand, SARS-CoV-2 infection is associated with an increased prevalence of acquired thrombophilia. Ferrari et al. reported on 89 consecutive patients hospitalized for COVID-19 infection and observed a 20% prevalence of S protein deficiency and a higher prevalence (72%) of antiphospholipid antibodies, mainly lupus anticoagulants. They concluded that acquired thrombophilia does not directly correlate with the occurrence of thrombotic events and is probably an integral part of the inflammation storm [98]. Another small study (19 subjects), focused on coagulation tests, showed increased levels of D-dimers, fibrinogen degradation products, fibrinogen and FVIII, and mildly lower activities of natural anticoagulant activities; ten patients tested positive for antiphospholipid antibodies, four of whom developed cerebral thrombotic events. These data suggest a triggering role for the simultaneous elevation of FVIII activity and the presence of antiphospholipid antibodies leading to thrombotic events [109].

Acute cerebrovascular disease has been reported in patients with severe COVID-19 and vascular risk factors [110]. The results of the study, conducted by Topcouglu et al., [111] showed that major thrombophilia did not exist in any of the studied stroke patients with COVID-19.

Since the state of hypercoagulation is directly correlated with COVID-19, we consider that studies on the genetic profiles of proteins involved in thrombophilia in patients who have had COVID-19 and thrombotic events are of great importance, both in treating and in preventing deaths due to COVID-19.

## 6. Conclusions

Given the evolution of patients infected with COVID-19 (some are asymptomatic, while others have severe symptoms and a rapid deterioration of their condition), there are still many unanswered questions about the mechanisms of action and consequences of the virus. Our work provides a structured and updated analysis of inherited thrombophilia and its involvement in COVID-19. No significant trends of predisposition to hereditary thrombophilia were apparent in COVID-19-positive patients who died of thrombotic complications. It was quite unexpected that patients who died, due to thrombotic complications, had no predisposition to congenital thrombophilia. We can conclude that hereditary thrombophilia can aggravate COVID-19 disease and is a marker of poor prognosis in the disease. Thus, we consider studies of the genetic profiles of proteins involved in thrombophilia in patients with COVID-19 and thrombotic events to be of great importance in both the treatment and prevention of deaths caused by COVID-19.

## Figures and Tables

**Table 1:** Complications associated with COVID-19.

Types of Disease	References
Cardiovascular	
Myocarditis	[29,30]
Acute myocardial infarction	[31]
Acute heart failure and cardiomyopathy	[31,32]
Dysrhythmias	[33]
Neurologic	
Guillain-Barré Syndrome	[34,35,36,37,38,39]
Encephalitis and encephalopathy	[40,41]
Acute cerebrovascular disease	[42]
Pulmonary	
Pneumothorax	[43,44,45,46,47,48,49]
Pneumomediastinum	[43,44,45,46,47,48]
Pulmonary mucormycosis	[50]
Pulmonary embolism	[51]
Rheumatologic	
Acute arthritis	[52]
Hepatic	
Acute liver failure	[53]
Neuropsychiatric	
Psychosis and suicidal behavior	[54,55,56,57]
Gastrointestinal	
Ischemic colitis	[58]
Dermatologic	
Digital ischemia	[59]
Chilblains	[60]

**Table 2:** Studies on hereditary thrombophilia in the era of COVID-19.

Evaluated Parameters	Comments	Author (Reference)
Activated partial thromboplastin time, prothrombin time, D-dimer, fibrinogen, protein C, protein S, antithrombin deficiencies, antiphospholipid antibodies	Although they reported a high prevalence of positive thrombophilia tests in COVID-19 infections, with a 20% prevalence of protein S deficiency, they are not correlated with the severity or prognosis of COVID-19.	Ferrari et al. [98]
Genes encoding antithrombin (*SERPINC1*), protein C and protein S	No thrombotic events were found in most subjects with severe thrombophilia during SARS-CoV-2 infection.	de la Morena-Barrio et al. [99]
Factor V Leiden, factor V 4070 A > G (Hr2), factor II G20210A, MTHFR C677 T, MTHFR A1298C, CBS 844ins68, PAI-1 4G/5G, glycoprotein IIIa T1565C (HPA-1a/b), ACE D/I, apolipoprotein E, AGT M235 T, ATR-1 A1166C, fibrinogen 455 G > A and factor XIII Val34Leu	One patient tested positive for apolipoprotein E e3/e4, heterozygous MTHFR A1298C, heterozygous ACE D/I, heterozygous AGT M235 T, and heterozygous FXIII Val34Leu.	Burlacu et al. [100]
Histological analysis and immunohistochemistry for a number of 41 patients who died of COVID-19	Increased alveolar damage and micro/macro vascular pulmonary thrombosis.	Bussani et al. [101]
Thromboelastography, D-dimer, fibrinogen, C-reactive protein, ferritin	Changes in fibrinogen, platelets and hypercoagulable thromboelastography profiles.	Yuriditsky et al. [102]
D-dimer, Alanine transaminase, C-reactive protein, erythrocyte sedimentation rate, partial thromboplastin time, white blood cell	Changes in the complete blood count, liver function test results, D-dimer levels, C-reactive protein, ferritin and coagulation panels.	Brosnahan et al. [103]
FV 506R/Q, MTHFR 223A/V, F2 20210G/A and PAI-1 4G/5G alleles	COVID-19 positive patients who died of thrombotic complications had no signs of predisposition to hereditary thrombophilia.	Abdullaev et al. [104]

## Data Availability

Not applicable.
